# Mid-term and long-term safety and efficacy of bioresorbable vascular scaffolds versus metallic everolimus-eluting stents in coronary artery disease: A weighted meta-analysis of seven randomised controlled trials including 5577 patients

**DOI:** 10.1007/s12471-017-1008-x

**Published:** 2017-06-13

**Authors:** J. Elias, I. M. van Dongen, R. P. Kraak, R. Y. G. Tijssen, B. E. P. M. Claessen, J. G. P. Tijssen, R. J. de Winter, J. J Piek, J. J. Wykrzykowska, J. P. S. Henriques

**Affiliations:** AMC Heartcenter, Academic Medical Center, University of Amsterdam, Amsterdam, The Netherlands

**Keywords:** Coronary artery disease, Percutaneous coronary intervention, Bioresorbable vascular scaffold, Stent, Device thrombosis, Meta-analysis

## Abstract

**Aims:**

Mid- and long-term safety and efficacy of the Absorb bioresorbable vascular scaffold (BVS) have been studied in randomised trials; however, most were not individually powered for clinical endpoints. We performed a weighted meta-analysis comparing mid- and long-term outcomes in patients treated with the BVS compared with the Xience metallic stent.

**Methods and results:**

Randomised trials comparing the BVS and Xience were identified by searching MEDLINE, EMBASE and conference abstracts. Seven trials were included (BVS *n* = 3258, Xience *n* = 2319) with follow-up between 1–3 years. The primary outcome of target lesion failure occurred more frequently in BVS compared with Xience [*OR* 1.34; 95% CI 1.11–1.62, *p* = 0.003]. Overall definite or probable device thrombosis occurred more frequently with the BVS [*OR *2.86; 95% CI 1.88–4.36, *p* < 0.001] and this extended beyond 1 year of follow-up [*OR* 4.13; 95% CI 1.99–8.57, *p* < 0.001]. Clinically indicated or ischaemia driven target lesion revascularisation [*OR* 1.43; 95% CI 1.11–1.83, *p* = 0.005] and myocardial infarction (all MI) [*OR* 1.64; 95% CI 1.20–2.23, *p* = 0.002] were more frequently seen in the BVS compared with Xience. Rates of target vessel failure [*OR* 1.15; 95% CI 0.91–1.46, *p* = 0.25] and cardiac death [*OR* 0.91; 95% CI 0.57–1.46, *p* = 0.71] were not significantly different between BVS and Xience.

**Conclusion:**

This meta-analysis shows a higher rate of target lesion failure and an almost threefold higher rate of device thrombosis in BVS compared with Xience, which extends beyond the first year. Device thrombosis did not lead to an overall increased (cardiac) mortality.

**Electronic supplementary material:**

The online version of this article (doi: 10.1007/s12471-017-1008-x) contains supplementary material, which is available to authorized users.

## Introduction

Bioresorbable scaffolds may theoretically overcome some limitations of current generation drug-eluting stents [1]. The Absorb (Abbott Vascular, Santa Clara, California, USA) bioresorbable vascular scaffold (BVS) is the most widely used bioresorbable device. Clinical trials enrolling patients with strict inclusion and exclusion criteria showed that the use of the BVS was safe and feasible with acceptable short- and mid-term clinical outcomes [2–7]. However, registries performed in more complex patients and lesions reported higher rates of early and late scaffold thrombosis [8–10]. In the ABSORB II trial, an ongoing risk of scaffold thrombosis up to 3 years of follow-up was observed [11]. The ABSORB III trial showed significantly higher 2‑year target lesion failure in the BVS compared with Xience [12], leading to an US Food and Drug Administration (FDA) warning [13]. The AIDA trial included a patient population reflecting routine clinical practice, and reported data earlier due to safety concerns. In AIDA, treatment with the BVS compared with Xience was associated with an increased incidence of device thrombosis throughout a median follow-up of 2 years [14]. A previous meta-analysis of randomised controlled trials comparing the BVS with Xience showed higher rates of scaffold thrombosis, mainly in the acute and subacute phase [15]. More recently, various trials also raised concerns on the use of the BVS with a longer follow-up, with a higher incidence of late and very late scaffold thrombosis [11, 12, 14, 16–20]. Sorrentino et al. performed a meta-analysis of randomised clinical trials with longer follow-up and raised the same concerns regarding the Absorb BVS; however, they did not perform an analysis on the events occurring beyond 1 year [21]. We performed a weighted meta-analysis on the available randomised controlled trials comparing mid- and long-term outcome (>12-month follow-up), together with a landmark analysis beyond 1 year of follow-up, in BVS compared with Xience metallic stent.

## Methods

### Search strategy and study selection

We searched PubMed, MEDLINE, and EMBASE for randomised studies on the BVS compared with Xience from inception to March 29th 2017. There were no language or other restrictions, and the results were only filtered on human studies. The search consisted of the following search terms including keywords and MESH terms for ‘Bioabsorbable’, ‘ABSORB’, ‘Bioresorbable stent’, ‘Everolimus’, ‘Controlled Clinical trial’ and ‘Randomized controlled trial’ (see the online Electronic Supplementary Material (ESM) for the complete MEDLINE search).

All retrieved studies were first screened independently by two of the investigators (JE and ID) at the title and/or abstract level. The remaining applicable studies were then reviewed in detail according to the following predefined inclusion and exclusion criteria. Inclusion criteria were randomised design, ≥50 enrolled patients and performance of an intention-to-treat analysis. Exclusion criteria were non-human studies or another comparison than of BVS versus Xience. If there were no published trial data on follow-up beyond 1 year, we searched for additional conference proceedings on long-term follow-up data of the particular trial.

### Risk of bias assessment and data extraction

Risk of bias in the included trials was assessed using the Cochrane risk of bias assessment tool in Review Manager (Version 5.3. Copenhagen, the Cochrane Collaboration, 2014), and data of the included trials were extracted by two of the investigators (JE and ID) [22]. Data were extracted from design papers, main papers, follow-up papers, conference proceedings and/or presentation slides, and combined in an Excel spreadsheet for further calculations where necessary.

### Study outcomes

The primary efficacy outcome was target lesion failure (TLF), a combined endpoint of cardiac death, target vessel myocardial infarction (TV-MI) and target lesion revascularisation (TLR). The primary safety outcome was definite or probable device thrombosis. Secondary outcomes were clinically indicated or ischaemia driven target lesion revascularisation (ID-TLR), the device oriented combined endpoint target vessel failure (TVF) consisting of cardiac death, target vessel MI (TV-MI) and target vessel revascularisation (TVR), as well as the occurrence of target vessel MI, all MI, all-cause death and cardiac death. All endpoints were defined according to the definitions of the original trials, and assessed according to the intention-to-treat analysis at the longest follow-up available. To assess long-term efficacy and safety we performed a landmark analysis after 1 year of follow-up comparing the rates of target lesion failure and device thrombosis occurring beyond 1 year of follow-up. For this landmark analysis patients were included in the denominator if they were free of the event of interest at 1‑year follow-up. Patients who withdrew informed consent, were lost-to-follow-up or died before 1‑year follow-up were excluded from the denominator.

### Statistical analysis

Continuous variables are reported as mean (±*SD*), categorical variables are expressed as *n/N* (%). The meta-analysis and statistical analysis for pooled odds ratios (*ORs*) was performed using the Peto fixed-effects model for categorical variables. Pooled *ORs* were calculated and are reported with 95% confidence intervals, a *p*-value <0.05 was considered statistically significant. Statistical heterogeneity was tested using the *χ* ^*2*^ test and the *I* ^*2*^ test. *I* ^*2*^ < 25% was considered to be low, 25–50% moderate, and >50% high heterogeneity. If no events occurred, the trial could not be added to the pooled *ORs*. All meta-analyses were performed on intention-to-treat basis and were performed with the Review Manager. To assess for publication bias, funnel plots were constructed for the primary outcomes. Additionally, we performed an influence analysis by omitting one trial at a time, and we carried out the Egger’s asymmetry test [23].

## Results

**Fig. 1:**
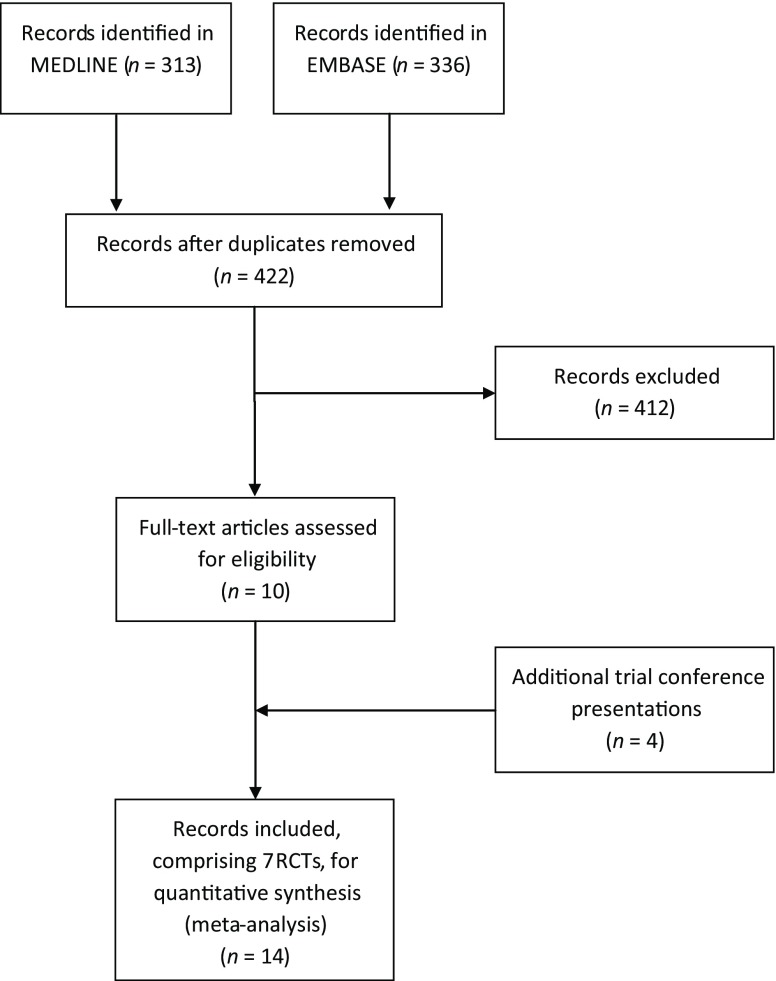
Search Flow Diagram

The search identified 422 publications. In total, seven eligible randomised controlled trials comparing BVS and Xience were identified after screening [11, 12, 14, 16–19]. For four trials the 2‑year outcomes were only available as conference proceedings [12, 17–19]. Fig. 1 depicts the flow diagram of the search; the MEDLINE search strategy and risk of bias assessment of the included trials are shown in the online ESM files. One trial had a third group of patients receiving a Biolimus-eluting stent; results from this patient group were excluded [17]. The ABSORB II trial started enrolment in 2011 and is depicted as ‘ABSORB II (2011)’ and so on. The primary objective of the original trials differed: three trials investigated clinical outcomes, three trials investigated late lumen loss on follow-up angiography and one trial investigated the optimal frequency domain imaging-derived healing score at 6 months. Follow-up duration also differed: one trial described a 3-year follow-up, five trials described 2‑year follow-up and one trial reported a median follow-up duration of two years [Q1-Q3: 507-895 days]. Definitions of target lesion failure, target lesion failure, and aspirin and dual antiplatelet therapy (DAPT) prescriptions differed between some of the trials. Table 1 of the online ESM shows all the relevant trial characteristics. The degree of bias within included trials was small (ESM Table 2).

### Patient and lesion characteristics

The seven trials included 5577 patients, randomised to either BVS (*n* = 3258) or Xience (*n* = 2319). The pooled mean age was 63 ± 10 years, with 66.7% of patients being male and 25.2% diabetic, of which 32% insulin-dependent. In total, 12.0% of patients presented with non-ST-elevated myocardial infarction (STEMI) on admission, 7.3% non-STEMI and 20.6% unstable angina pectoris. Periprocedural DAPT was administered in 98.9%. Table 1 shows the baseline characteristics of the patients and lesions included in the trials.

**Table 1:** Patient and lesion characteristics per included trial

	ABSORB II (2011)	EVERBIO II (2012)	ABSORB China (2013)	ABSORB Japan (2013)	AIDA (2013)	ABSORB III (2013)	TROFI II (2014)
**Patient characteristics**							
Randomised, *n*	501	240	480	400	1854	2008	191
Included, *n*	501	158	475	400	1845	2008	191
Age (years)	61 ± 10	65 ± 11	57 ± 11	67±9	64 ± 11	64 ± 11	59 ± 10
Men	385 (77)	125 (79)	343 (72)	309 (77)	1370 (74)	1415 (70)	157 (82)
Diabetes	120 (24)	30 (19)	115 (24)	144 (36)	324 (18)	640 (32)	32 (17)
– Insulin dependent	36 (7)	5 (3)	41 (9)	35 (9)	110 (6)	215 (11)	8 (4)
Dyslipidaemia	385 (77)	94 (5)	192 (40)	328 (82)	694 (38)	1732 (86)	115 (6)
ACS at admission	105 (21)	55 (35)	306 (64)	48 (12)	1029 (56)	523 (26)	191 (100)
– STEMI	0	15 (9)	0	0	465 (25)	0	191 (100)
– NSTEMI	0	29 (18)	0	0	377 (20)	0	0
– UAP	105 (21)	11 (7)	306 (64)	48 (12)	157 (9)	523 (26)	0
DAPT^a^	472 (94)	158 (100)	468 (99)	400 (100)	1845 (100)	1987 (99)	191 (100)
– Clopidogrel or Ticlopidine	427 (90)	NA	465 (99)	400 (100)	645 (35)	1268 (64)	65 (34)
– Prasugrel or Ticagrelor	45 (20)	NA	3 (1)	0	1198 (65)	719 (36)	127 (66)
**Lesion characteristics**							
Randomised, *n*	546	208	503	412	2446	2098	193
Diameter stenosis (%)	59.0 ± 11.3	80.5 ± 15.7	64.9 ± 0.82	64.6 ± 11.1	NA	65.5 ± 12.2	89.7 ± 15.3
RVD (mm)	2.60 ± 0.39	2.57 ± 0.65	2.81 ± 0.03	2.74 ± 0.45	3.05 ± 0.42	2.66 ± 0.45	2.81 ± 0.50
Length (mm)	13.8 ± 11.4	NA	14.0 ± 0.31	13.4 ± 5.36	19.0 ± 9.25	12.8 ± 5.5	13.1 ± 7.2
Type B2/C	254 (47)	67 (32)	369 (74)	313 (76)	1288 (53)	1462 (70)	NA

Overall baseline patient and lesion characteristics per included trial. Data are presented as number (%) and continuous data as mean (±SD). ACS acute coronary syndrome, STEMI ST-elevated myocardial infarction, NSTEMI non-ST-elevated myocardial infarction, UAP unstable angina pectoris, DAPT dual anti-platelet therapy, RVD reference vessel diameter

^a^Peri-procedural

In total, 6406 lesions were included in the trials of which 58% were randomised to BVS implantation. Pre-dilatation was performed in 97.7% of BVS, and in 93.5% of Xience-treated lesions. Post-dilatation was performed in 66.7% of BVS, and in 50.3% of Xience-treated patients. Device success, reported in six trials, was achieved in 96.3% of BVS, and in 99.4% of Xience patients. Table 2 depicts all available device implantation characteristics.

**Table 2:** Device implantation characteristics

	ABSORB II (2011)		EVERBIO II (2012)		ABSORB China (2013)		ABSORB Japan (2013)		AIDA (2013)		ABSORB III (2013)		TROFI II (2014)	
	Absorb	Xience	Absorb	Xience	Absorb	Xience	Absorb	Xience	Absorb	Xience	Absorb	Xience	Absorb	Xience
Pre-dilatation	364 (100)	180 (99)	93 (97)^a^	96 (86)	250 (99.6)	247 (98.0)	275 (100)	137 (100)	1199^a,c^ (97)	1103^c^ (91)	NA	NA	53 (55.8)	50 (51.0)
Pre-dilatation balloon size	NA	NA	NA	NA	2.8 ± 0.4	2.7 ± 0.4	2.80 ± 0.37	2.86 ± 0.36	2.71 ± 0.38^a^	2.64 ± 0.38	NA	NA	NA	NA
Pre-dilatation maximum pressure	NA	NA	NA	NA	12.4 ± 3.3^a^	11.8 ± 3.3	11.6 ± 3.8	11.9 ± 3.7	11.8 ± 3.0^a^	11.3 ± 3.0	NA	NA	NA	NA
Nominal size scaffold/stent	3.01 ± 0.31	3.05 ± 0.28	3.1 ± 0.4	3.0 ± 1.0	3.1 ± 0.4	3.1 ± 0.4	3.09 ± 0.37	3.13 ± 0.38	3.07 ± 0.37	3.05 ± 0.40	3.18 ± 0.43^a^	3.12 ± 0.45	3.25 ± 0.30^a^	3.12 ± 0.37
Implantation maximum balloon pressure	14.23 ± 3.43^b^	15.03 ± 3.33^b^	13.6 ± 2.8^a^	14.6 ± 2.9	12.8 ± 2.4	12.8 ± 2.8	10.4 ± 3.0^a^	11.2 ± 2.7	13.0 ± 2.7^a^	13.5 ± 2.7	15.4 ± 3.0	15.4 ± 3.2	14.1 ± 3.8	13.3 ± 3.0
Post-dilatation	221 (61)	107 (59)	33 (34)	35 (31)	162^a,c^ (63)	141^c^ (54.4)	226^c^ (82.2)	106^c^ (77.4)	915^c^ (74)^a^	594^c^ (49)	866^a^ (65.5)	351 (51.2)	48^a^ (50.5)	25 (25.5)
Post-dilatation balloon size	3.08 ± 0.34^b^	3.16 ± 0.36^b^	NA	NA	3.3 ± 0.4	3.2 ± 0.4	3.18 ± 0.44^a^	3.29 ± 0.51	3.28 ± 0.44	3.29 ± 0.49	NA	NA	3.51 ± 0.34	3.29 ± 0.62
Post-dilatation with non-compliant balloon	NA	NA	NA	NA	NA	NA	NA	NA	NA	NA	NA	NA	43^a^ (89.6)	13 (52.0)
Post-dilatation maximum balloon pressure	^b^	^b^	NA	NA	16.8 ± 3.8	16.9 ± 3.4	15.5 ± 4.2	16.0 ± 3.9	15.4 ± 3.8	15.6 ± 3.5	NA	NA	15.8 ± 3.4^a^	18.6 ± 3.9
Post-dilatation balloon >0.5 mm	^b^	^b^	NA	NA	NA	NA	9^c^ (4.0)	NA	18/912 (2)	14/593 (2)	NA	NA	NA	NA
Device success	99%	100%	NA	NA	98%	99.6%	98.9%	99.3%	92%^a^	98%	94.3%^a^	99.3%	95.8%	100%

Available device implantation characteristics and device success per included trial. Data are presented as number (%) and continues data as mean (±SD)

^a^Significantly different from Xience group

^b^Implant or post-dilatation

^c^Per treated lesion

*NA* not applicable

### Primary and secondary endpoints

**Fig. 2:**
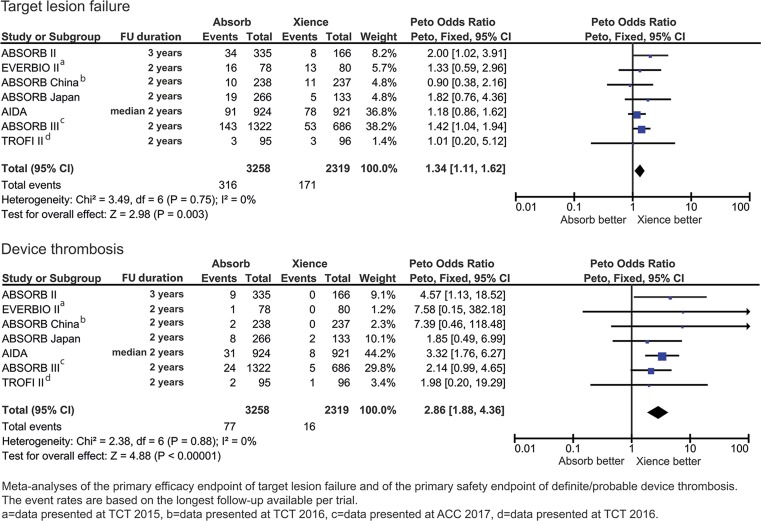
Meta-analyses of the primary efficacy endpoint of target lesion failure and the primary safety endpoint of device thrombosis at longest follow-up available

The primary outcome of target lesion failure occurred significantly more often with the BVS [*OR* 1.34; 95% CI 1.11–1.62, *p* = 0.003]. Definite or probable stent thrombosis occurred significantly more frequently in patients treated with the BVS (crude rates; BVS 2.4%, Xience 0.7% [*OR* 2.86; 95% CI 1.88–4.36, *p* < 0.001]) (Fig. 2). Timing of scaffold and stent thrombosis events differed between the two devices. Early device thrombosis occurred in 34 of BVS and in 11 of Xience-treated patients, late device thrombosis in 17 of BVS and in 2 of Xience-treated patients, and very late in 26 of BVS and 3 of Xience-treated patients (Table 3). ESM Fig. 3 depicts the funnel plots of both primary endpoints. Visual estimation of the funnel plots did not suggest any important influence of small studies on the primary study outcomes, nor did the Egger’s asymmetry tests for target lesion failure (intercept 0.117 [95% CI −1.539–1.772], two-sided *p*-value of 0.863) and definite/probable device thrombosis (intercept 0.322 [95% CI −0.936–1.580], two-sided *p*-value of 0.539). The influence analysis showed that by omitting every trial, the total ORs did not alter direction (ESM Tables 3 and 4).

**Table 3:** Timing of stent or scaffold thrombosis cases per included trial

	ABSORB II (2011)		EVERBIO II (2012)		ABSORB China (2013)		ABSORB Japan (2013)		AIDA (2013)		ABSORB III (2013)		TROFI II (2014)	
	Absorb (335)	Xience (166)	Absorb (78)	Xience (80)	Absorb (238)	Xience (237)	Absorb (266)	Xience (133)	Absorb (924)	Xience (921)	Absorb (1322)	Xience (686)	Absorb (95)	Xience (96)
Early ST (0–30 days)	2	0	0	0	1	0	3	1	13	5	14	5	1	0
Late ST (31 days–1 year)	1	0	1	0	0	0	1	1	8	1	6	0	0	0
Very late ST (>1 year)	6	0	0	0	1	0	4	0	10	2	4	0	1	1

Cases of definite or probable stent/scaffold thrombosis (ST) divided over early, late and very late time points after the index procedure. *ST* scaffold or stent thrombosis

Secondary outcomes showed that clinically indicated or ischaemia driven target lesion revascularisation, all MI and target vessel MI occurred significantly more frequently with the BVS. The rate of target vessel failure was not significantly different between BVS and Xience. All-cause death [*OR* 0.70; 95% CI 0.48–1.03, *p* = 0.07] and cardiac death [*OR* 0.91; 95% CI 0.57–1.46, *p* = 0.71] were also non-significantly different between the BVS and Xience (Fig. 3). Heterogeneity was low for all outcomes (0–7%).

**Fig. 3:**
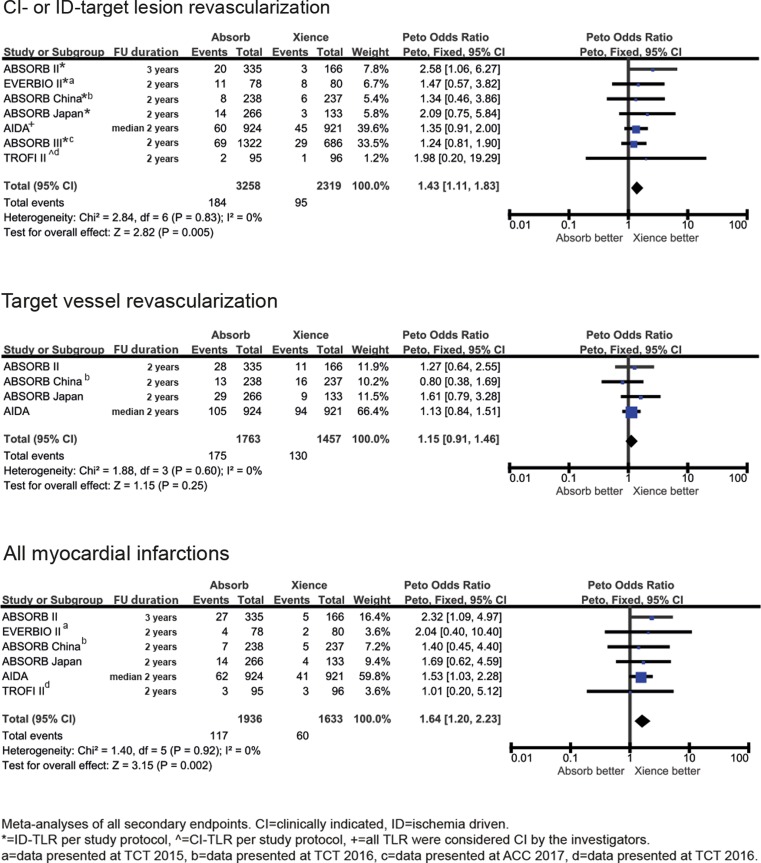
Meta-analyses of all secondary endpoints at longest follow-up available

In ESM Figs. 1 and 2, forest plots of the primary and secondary endpoints at 1 year (for EVERBIO II at 9‑month follow-up and for TROFI II at 6‑month follow-up) are depicted. At 1‑year follow-up, definite or probable device thrombosis, all MI and target vessel MI occurred significantly more frequently with the BVS: *OR* 2.43 [95% CI 1.45–4.04, *p* < 0.001], *OR* 1.39 [95% CI 1.06–1.82, *p* = 0.02] and *OR* 1.48 [95% CI 1.09–2.01, *p* = 0.01] respectively. All other endpoints at 1‑year follow-up were not significantly different between the two treatment groups.

Fig. 4 shows the number of the primary outcome events and associated odds ratios beyond 1 year of follow-up (for EVERBIO II beyond 9 months of follow-up and for TROFI II beyond 6 months of follow-up). Occurrence of target lesion failure events after 1 year occurred significantly more frequently in the BVS patients [*OR* 1.55; 95% CI 1.12–2.14, *p* = 0.008] and device thrombosis after 1‑year follow-up occurred in 27 patients treated with the BVS and in 3 patients treated with Xience [*OR* 4.13; 95% CI 1.99–8.57, *p* < 0.001]. During all time periods (early, late and very late) device thrombosis rates were higher in the BVS group (Fig. 5).

**Fig. 3 (continued):**
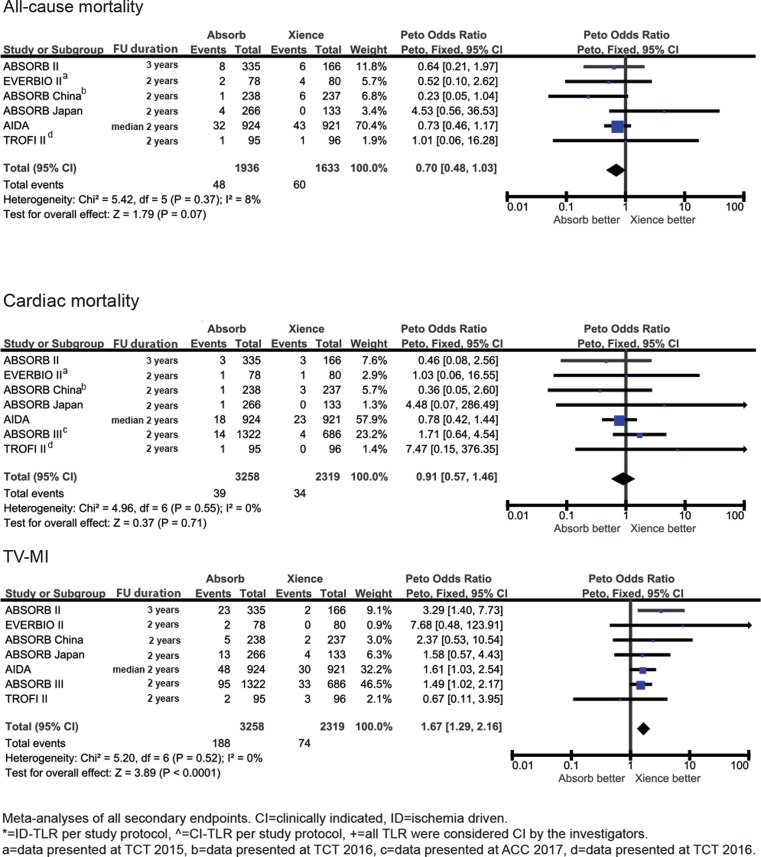
Meta-analyses of all secondary endpoints at longest follow-up available

**Fig. 4:**
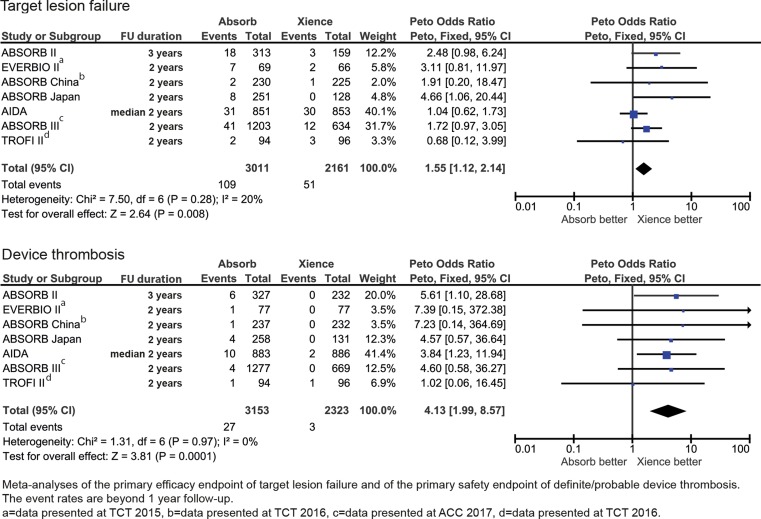
Meta-analyses of the primary efficacy and safety endpoints beyond one year follow-up

**Fig. 5:**
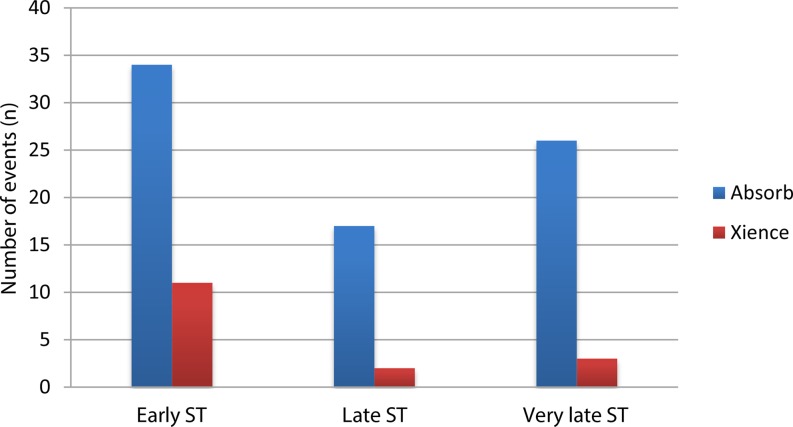
Number of events of early, late and very late device thrombosis between Absorb and Xience

Considering 4 of the 7 randomised trials did not publish their long-term data, we also performed a meta-analysis of the primary endpoint using only the published trials (*n* = 3). However, excluding these trials did not lead to a major difference in primary outcomes (target lesion failure: *OR* 1.34; 95% CI 1.02–1.76, *p* = 0.03; definite or probable device thrombosis: *OR* 3.17; 95% CI 1.87–5.38, *p* < 0.001) (ESM Fig. 4).

## Discussion

This meta-analysis showed a higher target lesion failure in BVS compared with the Xience. Also, in BVS, a highly significantly increased risk for definite and probable device thrombosis was observed compared with Xience. The incidence of all-cause mortality and cardiac death was not significantly different between the two groups.

Our meta-analysis demonstrates an ongoing higher risk of device thrombosis throughout the follow-up. Acute and subacute scaffold thrombosis has been attributed to inadequate DAPT and suboptimal implantation techniques of the BVS. One of the most intriguing findings of this meta-analysis is the ongoing and increased risk for definite or probable device thrombosis beyond 1 year of follow-up. These late and very-late scaffold thromboses are probably associated with different mechanisms and may be associated with resorption-related scaffold discontinuity and dismantling. Factors affecting flow conditions, such as (late-acquired) malapposed and uncovered struts due to heterogeneous endothelialisation of the scaffold, have also been suggested as potential causes [24, 25].

A specific BVS implantation protocol has been proposed to reduce the rates of scaffold thrombosis. This protocol consists of adequate pre-dilatation, correct sizing and post-dilatation (PSP) [26]. While this suggests that improved implantation techniques can prevent early device thrombosis, the effect of implantation techniques on long-term outcomes is less clear. Nevertheless, it is important to point out that post-dilatation was only performed in about 50% of the patients included in previous studies with infrequent use of intracoronary imaging [27]. In the studies included in this meta-analysis post-dilatation was only performed in 65–80% of the patients. Furthermore in a separate analysis performed by Stone pooling of previous ABSORB studies only 60.1% received predilatation and just 12.4% of patients received adequate high-pressure postdilatation with a noncompliant balloon [28].

The duration of DAPT is potentially associated with the occurrence of late scaffold thrombosis. The AIDA trial investigators very recently recommended continuation of DAPT for all BVS patients until 3 years post index PCI [14]. This recommendation is supported by the results from the DAPT trial: treatment with metallic drug-eluting stents and DAPT beyond 1 year compared with aspirin alone was associated with a significantly reduced risk of stent thrombosis and cardiovascular events [29]. Optimal DAPT duration for patients treated with BVS is unknown and might be challenging to determine given the variation in resorption time in every patient and lesion type. Furthermore, it is currently unknown if prolongation of DAPT will prevent late and very-late scaffold thrombosis.

Although the early and late thrombotic events in patients treated with the BVS are associated with worse outcome, these events did not translate into an overall higher mortality when compared with Xience. The bioresorbable technology holds great theoretical benefits, which can be expected to occur several years after implantation of the scaffold when the scaffold is completely dissolved. However, the BVS failed to demonstrate superiority in terms of vasomotion and did not meet non-inferiority in terms of late lumen loss [11, 16]. Therefore the suggested advantages of the BVS still need to be established. However, with current safety issues completing the ongoing future trials, COMPARE ABSORB and ABSORB IV might be challenging; nevertheless, long-term follow-up of these trials will shed additional light on the use of the first-generation BVS in more-complex patients.

Our study has several limitations: most of the trials included in this meta-analysis enrolled highly selected, mainly stable patients with non-complex lesions. Therefore generalising the results to all-comer populations should be done with caution. Also, our meta-analysis only included studies comparing the Absorb BVS to the Xience stent, therefore the results do not apply to other ‘bioresorbable stents’. None of the randomised trials were adequately powered for differences in individual clinical endpoints or for relatively rare endpoints. Furthermore, all the included trials used different event definitions and no patient-level data were available to examine predictors of worse outcome. There was only one study that reported a 3 year follow-up, other trials had a follow-up of 2 years or less, one trial reported median follow-up of 2 years. Since the resorption process of the scaffold is probably not completed at that time, extended follow-up is needed to fully assess the possible effect of the dissolving BVS on clinical outcomes.

In conclusion, this meta-analysis shows a significantly higher rate of target lesion failure and an almost threefold higher rate of device thrombosis in BVS compared with Xience metallic stent. This led to an increased incidence of MI, but not to an overall increased mortality. Further extension of follow-up will be essential to determine very long term clinical effects after full resorption of the first generation coronary bioresorbable scaffold.

## Caption Electronic Supplementary Material

ESM-Caption 1: Meta-analyses of the primary efficacy endpoint of target lesion failure and the primary safety endpoint of device thrombosis at maximum 1 year follow-up

ESM-Caption 2: Meta-analyses of all secondary endpoints at maximum 1 year follow-up

ESM-Caption 3: Funnel plots of both primary efficacy and safety endpoint at longest follow-up available

ESM-Caption 4: Primary endpoints TLF and definite/probable device thrombosis at longest follow-up available of published trails only
