# Supplementary material for: Mid-term and long-term safety and efficacy of bioresorbable vascular scaffolds versus metallic everolimus-eluting stents in coronary artery disease: A weighted meta-analysis of seven randomised controlled trials including 5577 patients
Source: Neth Heart J. 2017 Jun 13;25(7-8):429–38. doi: 10.1007/s12471-017-1008-x (PMC5513992; doi:10.1007/s12471-017-1008-x)
Supplement: Supplementary file 1 — ESM-Caption 1: Meta-analyses of the primary efficacy endpoint of target lesion failure and the primary safety endpoint of device thrombosis at maximum 1 year follow-up [file 12471_2017_1008_MOESM1_ESM.doc]

## Supplementary files

| **Supplementary table 1. Trial characteristics** | | | | | | | |
| --- | --- | --- | --- | --- | --- | --- | --- |
|  | **ABSORB II**  **(2011)** | **EVERBIO II (2012)** | **ABSORB China (2013)** | **ABSORB Japan (2013)** | **AIDA**  **(2013)** | **ABSORB III**  **(2013)** | **TROFI II**  **(2014)** |
| **Trial design** | International multi-center trial  2:1 Absorb BVS and Xience randomization | Single-center superiority trial  1:1:1 randomization Xience, BES, Absorb BVS | National multi-center non-inferiority trial  1:1 Absorb BVS and Xience randomization | National multi-center non-inferiority trial  2:1 Absorb BVS and Xience randomization | National multi-center non-inferiority trial  1:1 Absorb BVS and Xience randomization | International multi-center non-inferiority trial  2:1 Absorb BVS and Xience randomization | International multi-center non-inferiority trial  1:1 Absorb BVS and Xience randomization |
| **Primary outcomes** | Vasomotion at 3 years (superiority)  Difference in MLD post-procedure and at 3 years (non-inferiority) | In-stent LLL at 9m | In-segment LLL at 1y on angiography | TLF at 1y | TVF, all MI and TVR at 2y | TLF at 1y | Optimal frequency domain imaging-derived healing score (OFDI-HS) at 6m |
| **Secondary outcomes** | IVUS evaluation, acute success and clinical outcomes | Angiographic evaluation at 9m  Clinical outcomes at 1y | Acute success, clinical and angiographic outcomes at 1y | Acute success, angiographic and clinical outcomes at 13m | Acute success and additional clinical outcomes at 2y | IVUS evaluation at 3y  Angina, all-revascularization, ID-TVR at 1y (powered)  Acute success and clinical outcomes at 30 and 180 days, and 1/2/3/4/5y FU | Acute success, angina status at 6m, clinical outcomes at 1/6m, 1/2/3y |
| **Definitions TLF and TVF used** | TLF= cardiac death, TV-MI, CI-TLR  TVF= cardiac death, all MI, CI-TVR | TLF (DOCE)= cardiac death, all MI, all TLR  TVF= not assessed | TLF= cardiac death, TV-MI, ID-TLR  TVF= cardiac death, TV-MI, ID-TVR | TLF= cardiac death, TV-MI, ID-TLR  TVF= cardiac death, all MI, ID-TVR | TLF= cardiac death, TV-MI, ID-TVR  TVF= cardiac death, TV-MI, ID-TVR | TLF= cardiac death, TV-MI and ID-TLR  TVF= not assessed | TLF (DOCE)= cardiac death, TV-MI, CI-TLR  TVF= not assessed |
| **Longest follow-up duration available** | 3 year | 2 year | 2 year | 2 year | 707 days [Q1-Q3: 507-895 days] | 2 year | 2 year |
| **Average DAPT duration per protocol** | Aspirin ≥ trial duration  DAPT ≥ 180 days post index procedure | Aspirin indefinitely  DAPT ≥ 6m | Aspirin ≥ 5y  DAPT ≥ 1y | Aspirin indefinitely  DAPT ≥ 1y | Aspirin indefinitely  DAPT ≥ 1y | Aspirin indefinitely  DAPT ≥ 1y | Aspirin  DAPT ≥ 1y |
| **DAPT at 1 year*** | 413 (82) | NA | 461 (97) | 381 (96) | 1574 (88) | 1900 (96) | 156 (82) |
| **DAPT at 2 year*** | 178 (36) | NA | NA | 203 (52) | 164 (17) | 1305 (66) | NA |
| Trial design characteristics, primary and secondary outcomes, definitions, longest available follow-up duration, and DAPT usage per included trial. BVS = bioresorbable scaffold, XIENCE = everolimus-eluting stent, BES = biolimus-eluting stent, MLD = mean lumen diameter, LLL = late lumen loss, TLF = target lesion failure, TVF = target vessel failure, TV = target vessel, MI = myocardial infarction, ID = ischemia-driven, CI = clinically indicated, TVR = target vessel revascularization, TLR = target lesion revascularization, DAPT = dual anti-platelet therapy.  *percentages are of the total number of patients that reached 1 and 2 year follow-up, respectively | | | | | | | |

## MEDLINE search

## (((((((trials, randomized clinical[MeSH Terms]) OR randomized clinical trial[Title/Abstract]) OR randomized controlled trial[Title/Abstract]) OR trial[MeSH Terms]) OR trial[Title/Abstract])) AND (((((((Drug-eluting stents[MeSH Terms]) OR Everolimus[MeSH Terms]) OR DES[Title/Abstract]) OR Everolimus*[Title/Abstract]) OR metallic[Title/Abstract]) OR xience[Title/Abstract]) OR stent[Title/Abstract])) AND (((((((ABSORB[Title/Abstract]) OR BVS[Title/Abstract]) OR bioresorbable[Title/Abstract]) OR bioabsorbable[Title/Abstract]) OR BRS[Title/Abstract]) OR scaffold*[Title/Abstract]) OR absorbable implants[MeSH Terms])

## Figure Legends:

## Supplementary figure 1: Meta-analyses of the primary efficacy endpoint of target lesion failure and the primary safety endpoint of device thrombosis at maximum 1 year follow-up.

Supplementary figure 2: Meta-analyses of all secondary endpoints at maximum 1 year follow-up.

## Supplementary figure 3: Funnel plots of both primary efficacy and safety endpoint at longest follow-up available.

| **Supplementary table 2. Risk of bias assessment** | | | | | | | |
| --- | --- | --- | --- | --- | --- | --- | --- |
|  | **ABSORB II**  **(2011)** | **EVERBIO II (2012)** | **ABSORB China (2013)** | **ABSORB Japan (2013)** | **AIDA**  **(2013)** | **ABSORB III (2013)** | **TROFI II**  **(2014)** |
| **Random sequence generation** | **Low risk** (centralized interactive voice-web-based service) | **Low risk**  (computer-generated random numbers) | **Low risk**  (interactive voice-response system or an interactive web-response system) | **Low risk**  (central randomization service) | **Low risk**  (centralized interactive voice-web-based system) | **Low risk**  (interactive voice-response system) | **Low risk**  (dedicated web-based software) |
| **Allocation concealment** | **Low risk** | **Unclear risk**  (sealed nontransparant numbered envelopes) | **Low risk** | **Low risk** | **Low risk** | **Low risk** | **Low risk** |
| **Blinding of participants** | **Low risk**  (single-blind) | **High risk**  (patients were not blinded) | **High risk**  (open label) | **Low risk**  (single-blind) | **Low risk**  (single-blind) | **Low risk**  (single-blind) | **Low risk**  (single-blind) |
| **Blinding of outcome assessment** | **Low risk**  (Independent CEC) | **Low risk**  (outcome assessors and data analysts were blinded to the intervention) | **Low risk**  (Independent CEC) | **Low risk**  (Independent CEC) | **Low risk**  (independent CEC) | **Low risk**  (Independent CEC) | **Low risk**  (independent CEC) |
| **Incomplete outcome data** | **Low risk**  (depicted in flow-chart) | **Low risk**  (depicted in flow-chart) | **Low risk**  (depicted in flow-chart) | **Low risk**  (depicted in flow-chart) | **Low risk**  (depicted in flow-chart) | **Low risk**  (depicted in flow-chart) | **Low risk**  (depicted in flow-chart) |
| **Selective reporting** | **Low risk**  (all pre-defined endpoints were assessed) | **Low risk**  (all pre-defined endpoints were assessed) | **Low risk**  (all pre-defined endpoints were assessed) | **Low risk**  (all pre-defined endpoints were assessed) | **Low risk**  (all pre-defined endpoints were assessed) | **Low risk**  (all pre-defined endpoints were assessed) | **Low risk**  (all pre-defined endpoints were assessed) |
| Risk of bias assessment using the Cochrane risk of bias screening tool in Review Manager. CEC=critical event committee. | | | | | | | |

| **Supplementary table 3. Influence analysis of the primary endpoint of TLF beyond one year FU.** | | | | | |
| --- | --- | --- | --- | --- | --- |
|  | **BVS** | | **EES** | | **Peto OR [CI]** |
|  | Events | Total | Events | Total |  |
| **Omitting ABSORB II** | 91 | 2698 | 48 | 2002 | 1.45 [1.03-2.05] |
| **Omitting EVERBIO II** | 102 | 2942 | 49 | 2095 | 1.48 [1.06-2.07] |
| **Omitting ABSORB China** | 107 | 2781 | 50 | 1936 | 1.54 [1.11-2.14] |
| **Omitting ABSORB Japan** | 101 | 2760 | 51 | 2033 | 1.46 [1.05-2.04] |
| **Omitting AIDA** | 78 | 2160 | 21 | 1308 | 2.02 [1.33-3.07] |
| **Omitting ABSORB III** | 68 | 1808 | 39 | 1527 | 1.47 [1.00-2.18] |
| **Omitting TROFI II** | 107 | 2917 | 48 | 2065 | 1.59 [1.15-2.21] |

| **Supplementary table 4**. **Influence analysis of the primary endpoint of definite/probable device thrombosis beyond one year FU.** | | | | | |
| --- | --- | --- | --- | --- | --- |
|  | **BVS** | | **EES** | | **Peto OR [CI]** |
|  | Events | Total | Events | Total |  |
| **Omitting ABSORB II** | 21 | 2826 | 3 | 2091 | 3.83 [1.69-8.65] |
| **Omitting EVERBIO II** | 26 | 3076 | 3 | 2246 | 4.05 [1.92-8.50] |
| **Omitting ABSORB China** | 26 | 2916 | 3 | 2091 | 4.05 [1.93-8.51] |
| **Omitting ABSORB Japan** | 23 | 2895 | 3 | 2192 | 4.07 [1.87-8.88] |
| **Omitting AIDA** | 17 | 2270 | 1 | 1437 | 4.35 [1.68-11.29] |
| **Omitting ABSORB III** | 23 | 1876 | 3 | 1654 | 4.07 [1.86-8.88] |
| **Omitting TROFI II** | 26 | 3059 | 2 | 2227 | 4.58 [2.15-9.76] |
