# Supplementary figures and images for: Mid-term and long-term safety and efficacy of bioresorbable vascular scaffolds versus metallic everolimus-eluting stents in coronary artery disease: A weighted meta-analysis of seven randomised controlled trials including 5577 patients
Source: Neth Heart J. 2017 Jun 13;25(7-8):429–38. doi: 10.1007/s12471-017-1008-x (PMC5513992; doi:10.1007/s12471-017-1008-x)

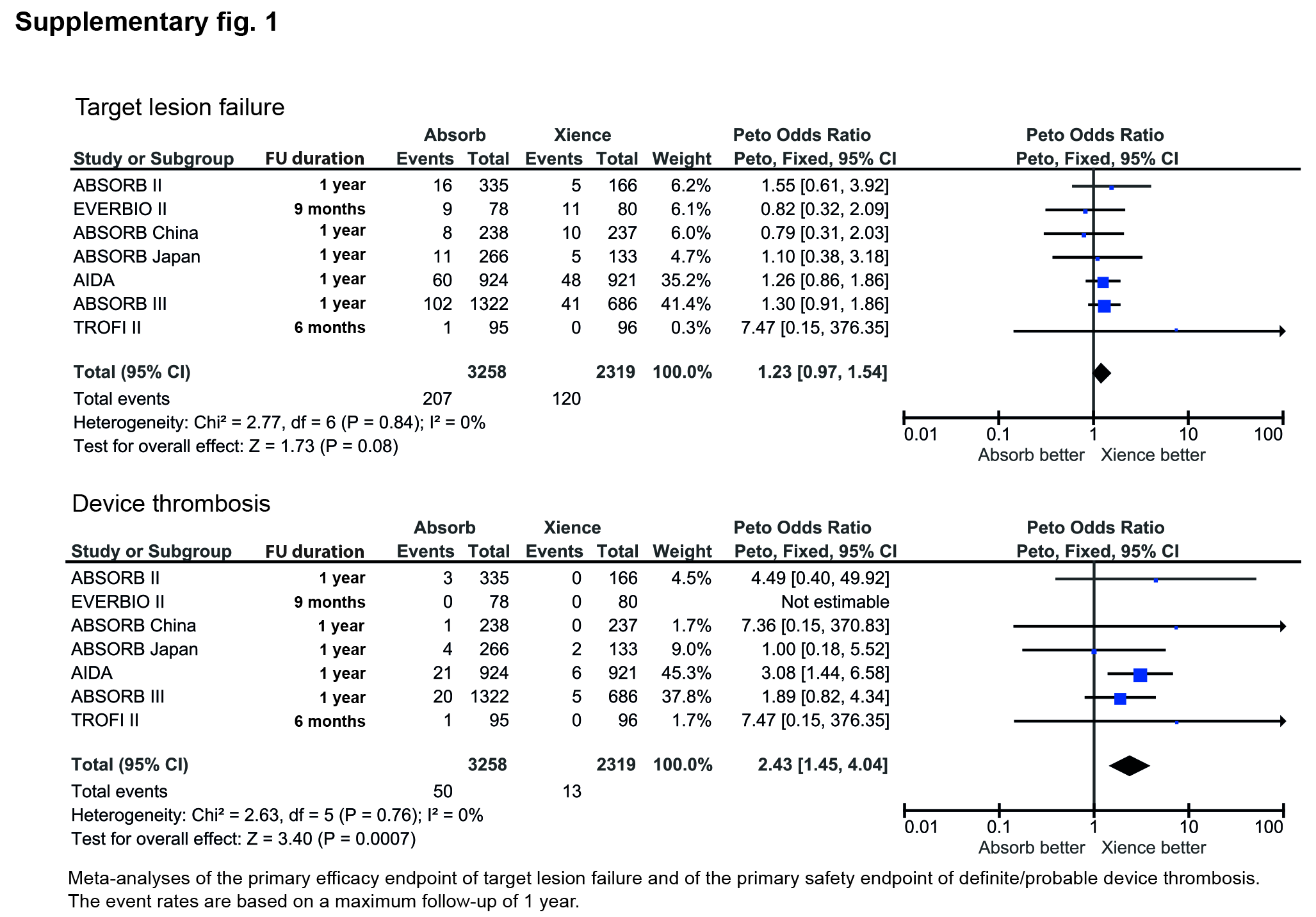

Supplement: Supplementary file 2 — ESM-Caption 2: Meta-analyses of all secondary endpoints at maximum 1 year follow-up [file 12471_2017_1008_MOESM2_ESM.tif]

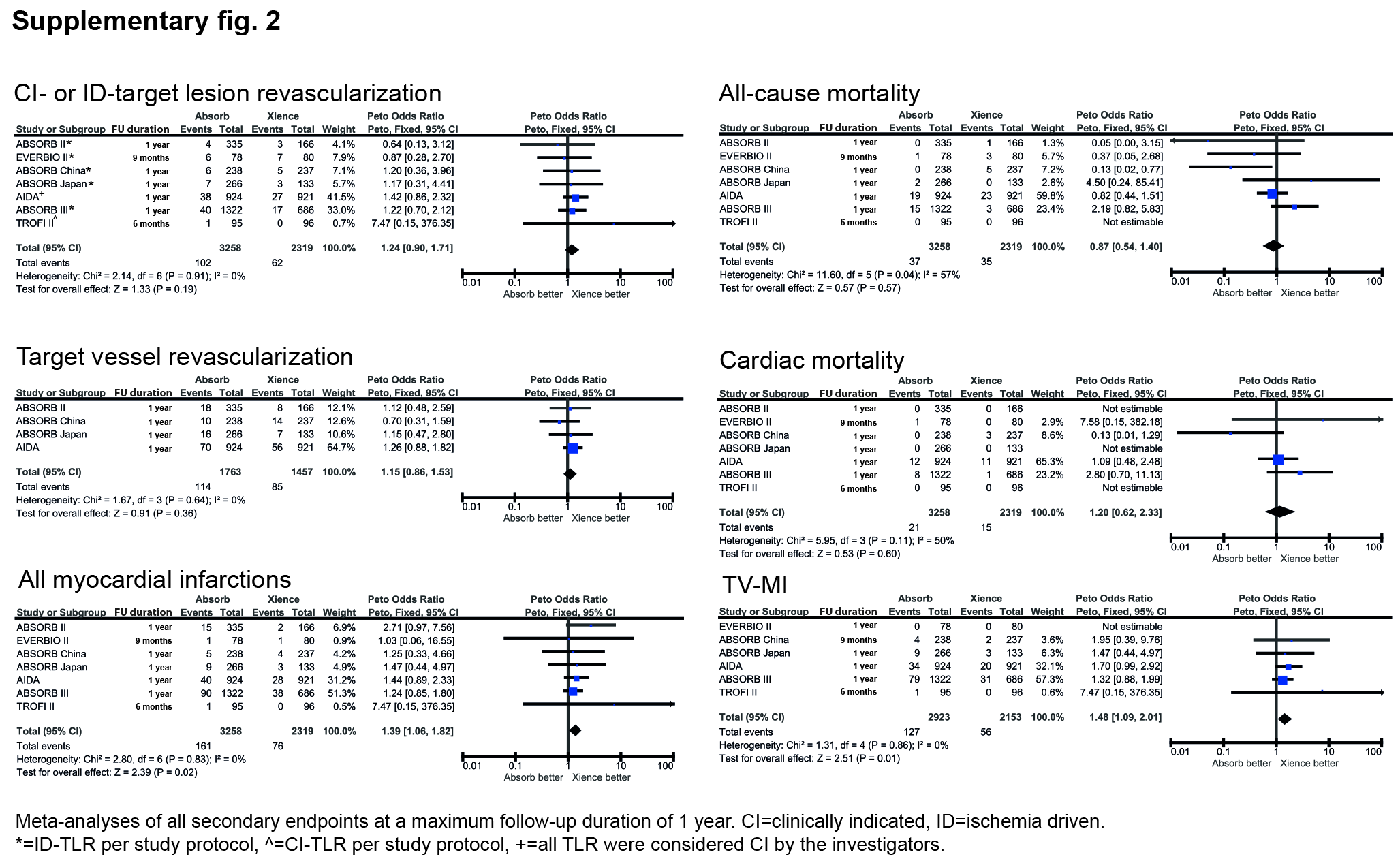

Supplement: Supplementary file 3 — ESM-Caption 3: Funnel plots of both primary efficacy and safety endpoint at longest follow-up available [file 12471_2017_1008_MOESM3_ESM.tif]

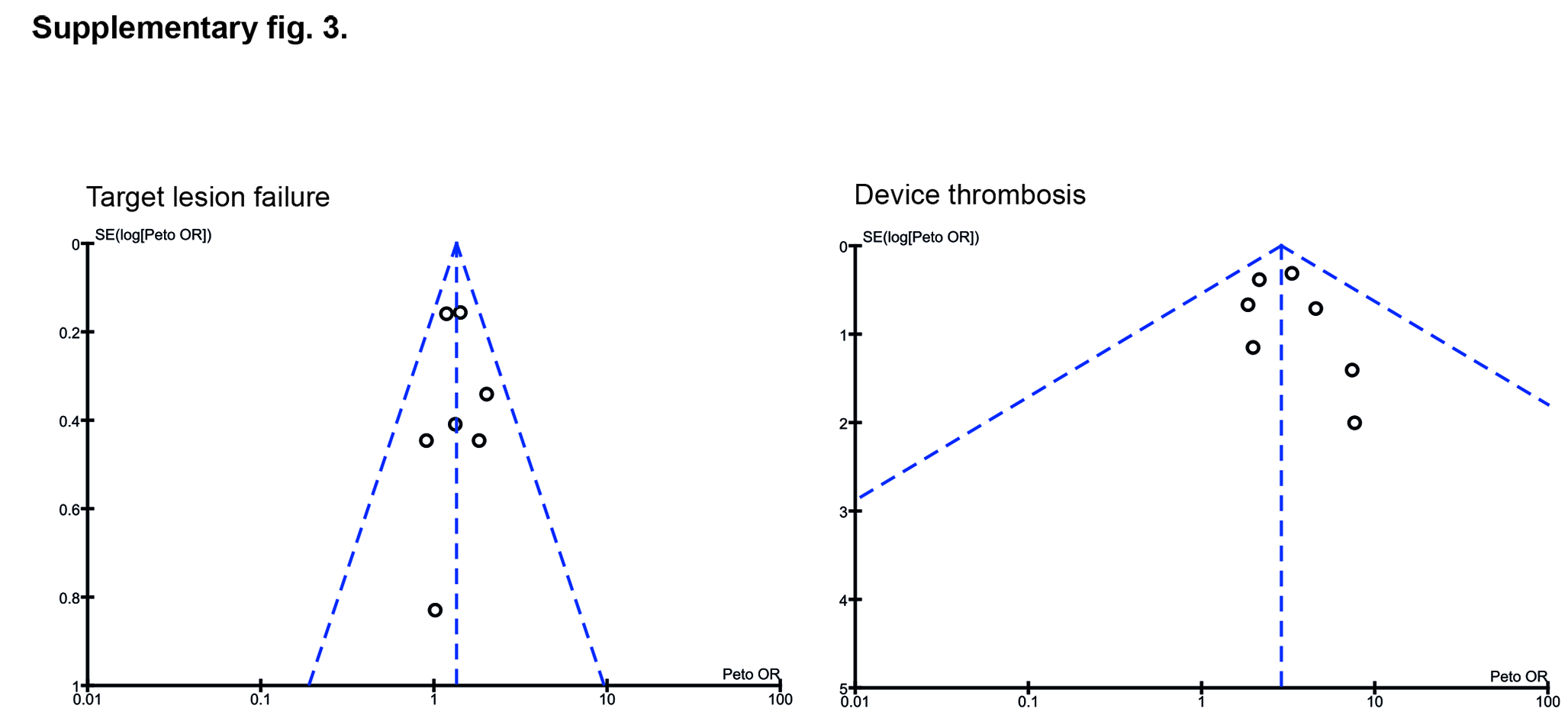

Supplement: Supplementary file 4 — ESM-Caption 4: Primary endpoints TLF and definite/probable device thrombosis at longest follow-up available of published trails only [file 12471_2017_1008_MOESM4_ESM.tif]
